# Development of a genome atlas for discriminating benign, preinvasive, and invasive lung nodules

**DOI:** 10.1002/mco2.644

**Published:** 2024-07-19

**Authors:** Peng Liang, Minhua Peng, Jinsheng Tao, Bo Wang, Jinwang Wei, Lixuan Lin, Bo Cheng, Shan Xiong, Jianfu Li, Caichen Li, Ziwen Yu, Chunyan Li, Jun Wang, Hui Li, Zhiwei Chen, Jian‐Bing Fan, Wenhua Liang, Jianxing He

**Affiliations:** ^1^ Department of Thoracic Surgery and Oncology the First Affiliated Hospital of Guangzhou Medical University, Guangzhou Institute of Respiratory Health, State Key Laboratory of Respiratory Disease, National Clinical Research Center for Respiratory Disease Guangzhou Guangdong China; ^2^ AnchorDx Medical Co., Ltd Guangzhou Guangdong China; ^3^ Department of Data Science Genomicare Biotechnology (Shanghai) Co., Ltd. Shanghai China; ^4^ Department of Data Science Shanghai CreateCured Biotechnology Co., Ltd. Shanghai China; ^5^ AnchorDx Inc. Fremont California USA; ^6^ Department of Pathology Southern Medical University Guangzhou Guangdong China

**Keywords:** EGFR/TP53 mutations, epigenetic regulation, genome atlas, lung adenocarcinoma, molecular pathogenesis, therapeutic targets

## Abstract

To tackle misdiagnosis in lung cancer screening with low‐dose computed tomography (LDCT), we aimed to compile a genome atlas for differentiating benign, preinvasive, and invasive lung nodules and characterize their molecular pathogenesis. We collected 432 lung nodule tissue samples from Chinese patients, spanning benign, atypical adenomatous hyperplasia (AAH), adenocarcinoma in situ (AIS), minimally invasive adenocarcinoma (MIA), and invasive adenocarcinoma (IA). We performed comprehensive sequencing, examining somatic variants, gene expressions, and methylation levels. Our findings uncovered EGFR and TP53 mutations as key drivers in ‐ early lung cancer development, with EGFR mutation frequency increasing with disease progression. Both EGFR mutations and EGF/EGFR hypo‐methylation activated the EGFR pathway, fueling cancer growth. Transcriptome analysis identified four lung nodule subtypes (G1‐4) with distinct molecular features and immune cell infiltrations: EGFR‐driven G1, EGFR/TP53 co‐mutation G2, inflamed G3, stem‐like G4. Estrogen/androgen response was associated with the EGFR pathway, proposing a new therapy combining tyrosine kinase inhibitors with antiestrogens. Preinvasive nodules exhibited stem cell pathway enrichment, potentially hindering invasion. Epigenetic regulation of various genes was essential for lung cancer initiation and development. This study provides insights into the molecular mechanism of neoplastic progression and identifies potential diagnostic biomarkers and therapeutic targets for lung cancer.

## Graphical abstract

EGFR and TP53 mutations were crucial for early lung cancers development. With EGF/EGFR hypo‐methylation accelerating cancer growth by activating EGFR pathway. Transcriptome‐based stratification of lung nodules revealed four subtypes (G1‐4) related to specific molecular features and immune cell infiltrations. Epigenetic regulation through an array of genes was essential for lung cancer initiation and development.

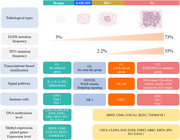

## 1 INTRODUCTION

Lung cancer is the most common malignancy and the primary cause of cancer‐related death worldwide.^1^ The risk factors for lung cancer include tobacco smoking, exposure to environmental chemicals, genetic predisposition, etc.^2^, ^3^ Early diagnosis is associated with a favorable prognosis in lung cancer, as evidenced by the significantly increased 5‐year overall survival rate for stage I (68% to 92%) compared to stage IV (< 10%).^4^

Currently, LDCT is extensively utilized for detecting small lung nodules that might be overlooked by conventional X‐ray.^5^ Lung nodules less than 30 mm in size pose a significant challenge in assessing their malignant potential, often leading to unnecessary diagnostic work‐ups.^6^ Adenocarcinoma is the predominant type of lung cancer. The most prevalent subset, lung adenocarcinoma (LUAD), is believed to progress stepwise from preinvasive lesions of atypical adenomatous hyperplasia (AAH), adenocarcinoma in situ (AIS), minimally invasive adenocarcinoma (MIA), to invasive adenocarcinoma (IA) with a lepidic growth pattern.^7^, ^8^ Although early screening of lung cancers through LDCT reduces mortality, the resulting high false‐positive rate and misdiagnosis emphasize the need for a deeper comprehension of the molecular mechanisms underlying benign, preinvasive, and invasive lung nodules. This understanding is crucial for discovering diagnostic biomarkers that can accurately identify early‐stage lung cancer.

Accumulated evidence from next‐generation sequencing studies has shed light on the distinct genetic characteristics of various subtypes of lung adenocarcinoma nodules.^9^, ^10^, ^11^, ^12^ Hu et al. have shown the multiregion exome sequencing of 116 AAH, AIS, MIA, and ADC (lung adenocarcinoma) samples, observing genomics evolution at both the single nucleotide level and chromosomal levels.^7^, ^9^ They also noted a progressive increase in methylation aberrations from AAH to AIS, MIA, and ADC, suggesting that global hypomethylation might influence chromosomal instability, mutagenesis and tumor immune microenvironment during precancer progression. Chen et al. have reported on the exome and transcriptome sequencing of preinvasive and invasive lung adenocarcinoma, identifying significantly mutated genes such as *EGFR* (Epidermal Growth Factor Receptor), *RBM10* (RNA binding motif protein 10), *BRAF* (B‐Raf proto‐oncogene), *ERBB2* (erb‐b2 receptor tyrosine kinase 2), *TP53* (tumor protein p53), *KRAS* (KRAS proto‐oncogene, GTPase), *MAP2K1*(mitogen‐activated protein kinase kinase 1), and *MET* (mesenchymal to epithelial transition factor) in the pre/minimally invasive group.^11^ Dejima et al. have characterized the immune contexture of invasive ADC and its precursors using transcriptomic immune profiling, T cell receptor sequencing, and multiplex immunofluorescence. Their findings demonstrated that anti‐tumor immunity evolved as a continuum with a gradually less effective and more intensively regulated immune response from AAH to AIS, MIA, and ADC.^12^ However, to fully understand the underlying biology and altered pathways involved in the malignancy development of lung nodules, comprehensive analyses are required. The use of systematic multiomics integrative approaches could significantly enhance our understanding of lung cancer stratification and facilitate the discovery of biomarkers for anti‐cancer drugs.^13^, ^14^

In this study, we aimed to distinguish between benign, preinvasive (AAH, AIS), and invasive (MIA, IA) nodules. To achieve this, we conduct a comprehensive analysis of data from whole exome sequencing (WES), transcriptomics, and DNA methylation sequencing of lung nodules samples from Chinese patients who had undergone surgical resection. Our findings offer a detailed genetic and epigenetic profile of benign nodules and early‐stage lung cancer, along with novel insights into the molecular mechanisms involved in the development of lung cancer.

## 2 RESULTS

### 2.1 Patient samples and characteristics

We collected 432 archived Formalin‐fixed paraffin‐embedded (FFPE) tissue samples from lung nodules. Samples with insufficient Genomic DNA (gDNA) amounts, low gDNA quality, low library yields, or failed sequencing quality control metrics were excluded. After thorough analysis, we obtained a final set of 422 samples, which were classified into three groups: benign (*n* = 124), preinvasive (AAH+AIS, *n* = 32), and invasive (MIA+IA, *n* = 266). The average age of the benign group was 50 years, younger than the preinvasive group (55 years) and the invasive group (57 years) (*p* < 0.05). In terms of gender, 59.4% of the preinvasive and invasive groups were female, while 62.1% of the benign group were male. Specifically, 78.2%, 90.6%, and 81.2% of the benign, preinvasive, and invasive groups were nonsmokers, respectively. Regarding nodule density, 51.9% of all nodules were solitary nodules (SNs), 37.9% were pure ground glass nodules (pGGNs), and 9.2% were part‐solid nodules (PSNs). Remarkably, 62.5% of preinvasive and 49.2% of invasive nodules were pGGNs, while only 7.3% of benign nodules were pGGNs. Additionally, 87.9% of benign patients had SNs, but this percentage decreased to 41.0% among the invasive groups. Among the benign nodules, 29.8% were inflammation, while 17.7% were tuberculosis. It is worth noting that 97.7% of invasive nodules were very early lung cancers, classified as American Joint Committee on Cancer (AJCC) stage I (Table 1).

**TABLE 1:** Clinical and demographic characteristics of patients.

Characteristics	Benign (*n* = 124)	Preinvasive (AAH+AIS) (*n* = 32)	Invasive (MIA+IA) (*n* = 266)	Total (*n* = 422)
**Age (mean ± SD)**	50.77 ± 13.41	55.31 ± 9.46	56.8 ± 11.44	54.92 ± 12.2
**Gender**				
Female	47 (37.9%)	19 (59.4%)	158 (59.4%)	
Male	77 (62.1%)	13 (40.6%)	108 (40.6%)	
**Smoking history**				
Nonsmokers	97 (78.2%)	29 (90.6%)	216 (81.2%)	342 (81.0%)
Smokers	27 (21.8%)	3 (9.4%)	50 (18.8%)	80 (19.0%)
**Nodule type**				
pGGN (G)	9 (7.3%)	20 (62.5%)	131 (49.2%)	160 (37.9%)
PSN (P)	6 (4.8%)	7 (21.9%)	26 (9.8%)	39 (9.2%)
SN (S)	109 (87.9%)	1 (3.1%)	109 (41.0%)	219 (51.9%)
NA		4 (12.5%)		4 (0.9%)
**Pathological Subtype**				
INF	37 (29.8%)			
TB	22 (17.7%)			
FUN	18 (14.5%)			
HAM	13 (10.5%)			
GRAN	8 (6.5%)			
FIBROSIS	7 (5.6%)			
PSP	7 (5.6%)			
other	12 (9.6%)			
IA			175 (65.8%)	
AIS		26 (81.3%)		
MIA			91 (34.2%)	
AAH		6 (18.8%)		
**AJCC stage**				
0		26 (81.3%)		
I			260 (97.7%)	
II			1 (0.4%)	
III			1 (0.4%)	
IV			3 (1.1%)	
NA		6 (18.8%)	1 (0.4%)	

Abbreviations: AAH, atypical adenomatous hyperplasia; AIS, adenocarcinoma in situ; FUN, fungus infection; GRAN, granulomas; HAM, hamartoma; IA, invasive adenocarcinomas; INF, inflammation; MIA, minimally invasive adenocarcinoma; NA, not applicable; pGGN, pure ground‐glass nodule; PSN, part solid nodule; PSP, pulmonary sclerosing pneumocytoma; SN, solid nodule; TB, tuberculosis.

### 2.2 Somatic mutation landscape of lung nodules

**FIGURE 1:**
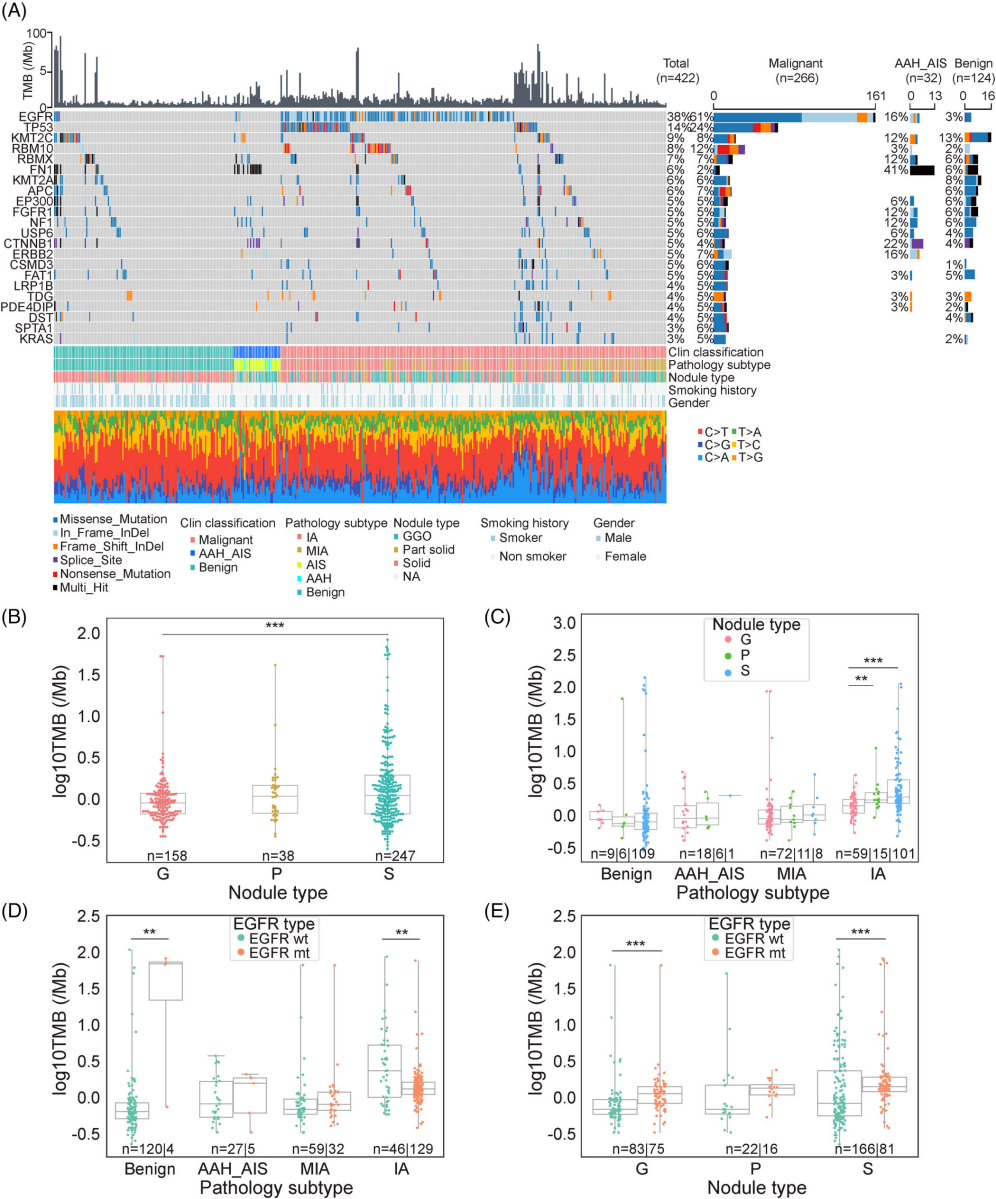
Landscape of somatic mutation in lung nodules. (A) Oncoprint of sequenced nodule samples including benign, AIS, AAH, MIA, and IA. Mutation type breakdowns are shown on the right. TMB is shown on the top. (B) Comparison of nodule types according to TMB. G: pure GGN; P: part‐solid nodule; S: solid nodule. (C) Comparison of pathological subtypes among nodule types, stratified by TMB. (D) Comparison of *EGFR* mutation status in different pathological subtypes, stratified by TMB. (E) Comparison of *EGFR* mutation status in different nodule types, stratified by TMB. In the boxplots, the upper and lower lines represent the first and third quartiles, and the center lines represent the median. ns, not significant, *p < 0.05; **p < 0.01; ***p < 0.001, Two‐sided Mann–Whitney *U* test.

In the WES study, we analyzed a total of 422 lung nodule tissues (124 benign, 32 AAH and AIS, 266 MIA and IA) and their matched white blood cells (WBCs). Twenty‐two significantly mutated genes were identified in the invasive samples (Figure 1A), among which the two most frequently mutated genes were *EGFR* (61%) and *TP53* (24%), consistent with previous reports.^11^

The frequency of *EGFR* mutations increased from benign (3%), to preinvasive (AAH+AIS, 16%), MIA (35%), and IA (73%) (Figure 1A). The *EGFR* mutation frequencies were statistically higher in females (vs. male, *p* = 0.0016) and nonsmokers (vs. smokers, *p* = 0.0004) (Figures 1A and S1). The most prevalent types of *EGFR* mutations identified were exon 21 p.L858R (39%) and exon19 deletions (36%) (Figure S2A), and these mutations were highly prevalent in MIA (81.25%, 50% exon 21 p. L858R, 31.25% exon 19 deletions) and IA (78.91%, 39.46% exon 21 p. L858R, 39.46% exon 19 deletions) (Figure S2B). These hotspots *EGFR* mutations observed in our early‐stage lung cancer cohort were consistent with those found in late‐stage lung cancers in another Chinese lung cancer cohort.^15^, ^16^ Other *EGFR* mutations, such as exon 20 insertions, exon 18 p.G719S/A, exon 18 p.E709A, exon 21 p.L861Q/R, and exon 20 p.T790M, were also detected (Figure S2A and B). Notably, *EGFR* mutations in benign nodules were infrequent and inactive, as demonstrated by functional annotation of mutated genes (Figure S6).

*TP53* mutations were significantly more prevalent in IA (35.43%) compared to MIA (2.2%) (Figure 1A) and were scarcely detected in preinvasive and benign samples. This suggests that *TP53* mutations might not influence nodule growth but could play an important role in promoting invasiveness development, which is in line with a recent finding.^17^ Patients harboring *TP53* mutations were more likely to be smokers, strongly associated with older age (≥ 55 years), male gender, solid nodules, and higher TMB (≥ 1.39/Mb) (Figure S1).

The mean TMB of this cohort was 3.34/Mb, which was lower than the studies of late‐stage lung cancers.^18^ The average TMB in invasive samples (MIA, IA) was 2.94/Mb. When comparing within nodule types, the TMB of solid nodules (S) was significantly higher than that of pure GGN (G) (*p* < 0.001) (Figure 1B). Furthermore, when TMB was compared within different pathological subtypes, we observed that it was higher in invasive stage compared to benign and preinvasive stage (Figure 1C). Interestingly, within the IA groups, the TMBs of pure GGN, part‐solid, and solid nodules gradually increased as the nodule density increased (Figure 1C). When intersecting pathological subtypes with *EGFR* mutation status, the median TMB in the *EGFR* mutation (MUT) group was significantly lower compared to the *EGFR* wild type (WT) group in IA (*p* < 0.01), consistent with a previous study.^19^ However, this trend was reversed among benign nodules, possibly due to the very low proportion of the *EGFR* MUT group in benign group (Figure 1D). We further compared *EGFR* mutation status among nodule types based on TMB and found that the median TMB in the *EGFR* mutation group was statistically higher than that in the *EGFR* WT group with pure GGNs (G) and solid nodules (S) (Figure 1E) (*p* < 0.001). Considering the impact of *TP*53 mutation on TMB in IA nodules, a group of *TP53* MUT & *EGFR* WT samples appeared to have the highest TMB compared with other combinations of *TP53* WT/MUT and *EGFR* WT/MUT (Figure S3), which is in agreement with other studies^16^, ^20^ showing that TMB was higher in tumors with *TP53* MUT or *EGFR* WT.

**FIGURE 2:**
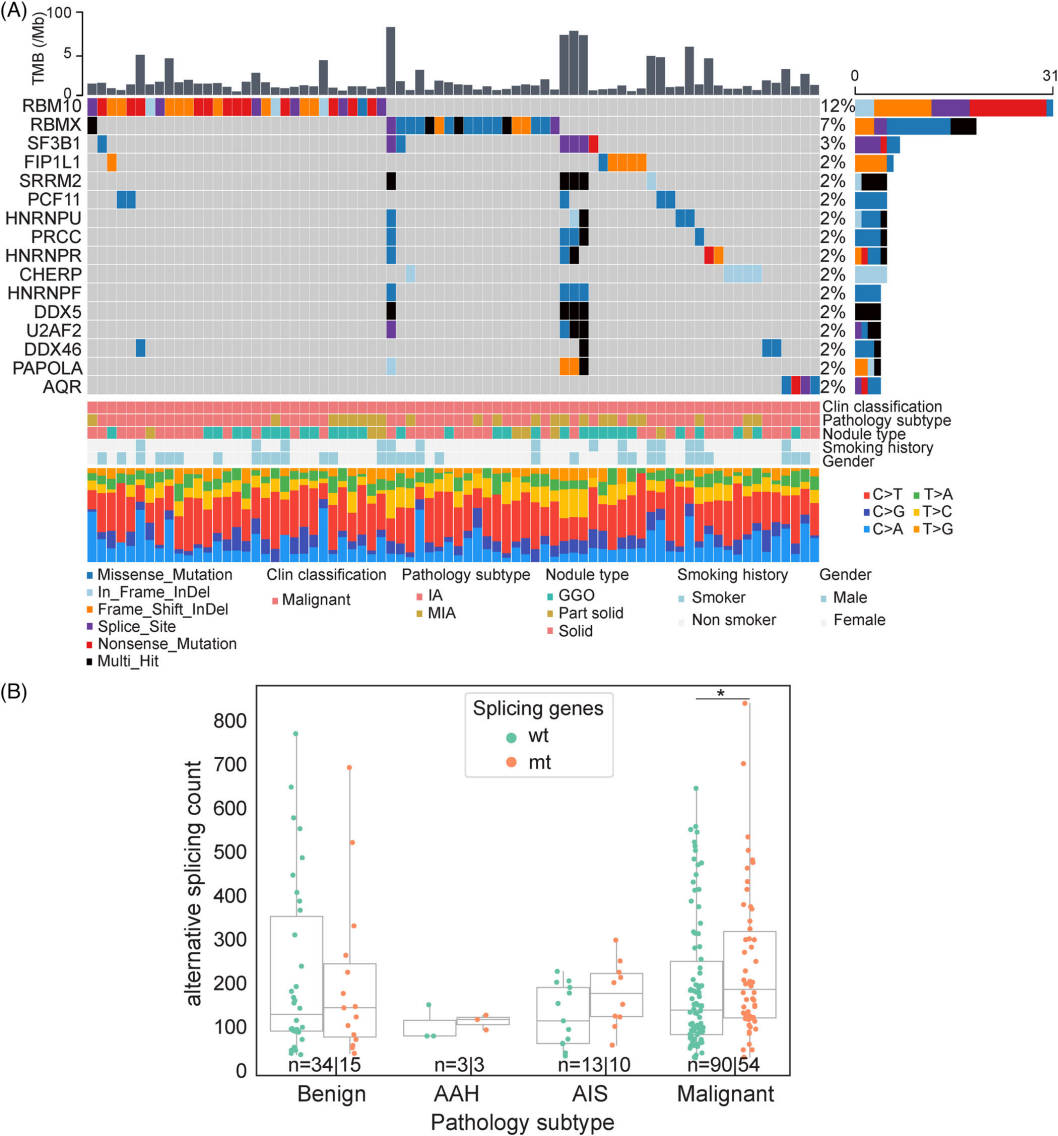
Landscape of Splicing Mutations in lung nodules. (A) Oncoplot of frequently splicing mutated genes in malignant nodules. The barplot above represents the TMB. The barplot on the right represents the type of mutations detected for each gene. (B) Comparison of alternative splicing events in different pathological subtypes of nodules with wild type or mutation in splicing genes. In the boxplots, the upper and lower lines represent the first and third quartiles, and the center lines represent the median. ns, not significant, *p < 0.05; **p < 0.01; ***p < 0.001, Two‐sided Mann–Whitney *U* test.

To assess the functional significance of mutated splicing genes, nodule samples were divided into splicing gene MUT and WT groups, and alternative splicing events from RNA‐Seq data were analyzed (Figure 2A). In invasive samples, a significantly higher number of splicing events were detected in samples carrying splicing gene mutation (Figure 2B). A total of 417 genes were identified with splicing events (splicing events ≥10). Pathway enrichment analysis revealed that these genes with splicing events were primarily involved in the cell cycle and other cancer‐related pathways, such as *KRAS* signaling and heme metabolism (Figure S4A). *RBM10* (12%) and *RBMX* (RNA Binding Motif Protein X‐Linked) (7%) were the most common splicing mutation genes. However, no significant difference in alternative splicing events was observed across pathological subtypes or between MUT and WT groups (excluding any splicing mutation) (Figure S4B and C).

Furthermore, we analyzed the detected CNVs by gene annotation based on the significance of differential genes between malignant and benign nodules. However, no lung cancer‐related gene was identified (data not shown).

### 2.3 The transcriptomic features and immune microenvironment of lung nodules

**FIGURE 3:**
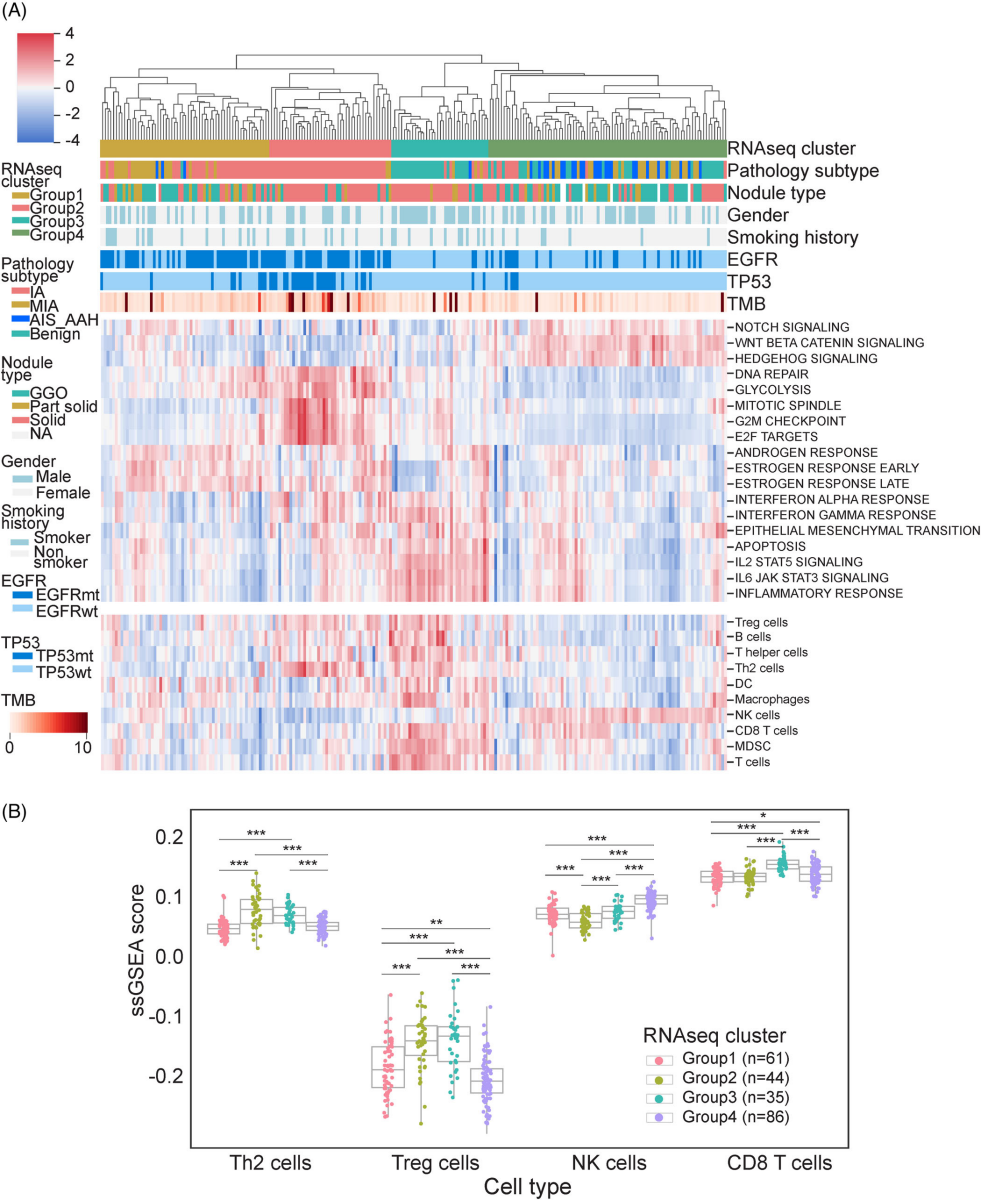
The transcriptomic features and immune microenvironment of lung nodules. (A) Phenotypes of RNA cluster subgroups. The cluster assignments of patients are shown on the top. The immune signature is on the bottom. (B) Immune cell comparison between RNA cluster subgroups.

To elucidate the transcriptomic landscape of lung nodules, RNA‐Seq was conducted on 226 lung nodules samples, comprising 52 benign, 29 AAH+AIS, and 145 MIA+IA cases. Through the integration of WES and RNA‐Seq data, four distinct molecular subgroups were identified based on unsupervised hierarchical clustering analysis of the gene expression profiles (Figure 3A).

Group 1, classified as the *EGFR*‐driven group, was predominantly composed of invasive nodules harboring *EGFR* mutations. Markedly increased expression of early and late estrogen response (ER) and androgen response (AR) genes was observed. Estrogen and its receptors have been postulated to play a role in the development and progression of lung cancer.^21^ Group 2, referred to as the *EGFR*/*TP53* co‐mutation group, was enriched for IA nodules carrying *EGFR*/*TP53* co‐mutations. These nodules exhibited upregulation of genes associated with mitotic spindles, the G2/M checkpoint, and E2F targets. Most of these genes are linked to cell cycle regulation and tumor proliferation. Notably, the glycolysis pathway was also upregulated in Group 2, aligning with the Warburg effect in tumor metabolism and supporting the idea that tumors generate energy through glycolysis to fuel proliferation and metastasis via metabolic reprogramming.^22^ Group 3, designated as the “inflamed group,” was enriched with benign nodules and included some MIA and IA samples. Increased expression of numerous inflammation‐related genes, such as *IL‐2* (Interleukin‐2), *IL‐6* (Interleukin‐6), and *IFN* (Interferons), was observed. Group 4 represented the stem‐like group, which comprised the majority of preinvasive (AAH, AIS) nodules and some MIA nodules. This group exhibited upregulation of stem cell pathways, including NOTCH, Wnt/β‐catenin, and hedgehog signaling, implicating the important role of stem cell pathways in tumor initiation (Figure 3A).

**FIGURE 4:**
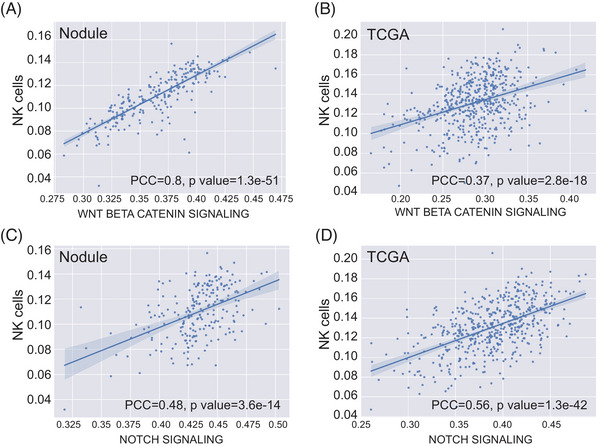
The correlation between NK cells and stem cell signaling pathway. (A‐B) The correlation between NK cells and WNT Beta Catenin signaling in lung nodules (A) or based on TCGA data (B). (C‐D) The correlation between NK cells and Notch signaling in lung nodules (C) or based on TCGA data (D). ns, not significant, *p < 0.05; **p < 0.01; ***p < 0.001, Two‐sided Mann–Whitney *U* test.

To explore the immune infiltration in lung nodules, we computed the immunophenotype score and immune cell infiltration score using single‐sample Gene Set Enrichment Analysis based on the RNA‐seq data, similar to a previous study.^23^ Notably, immune cell infiltration seemed more pronounced in the inflamed (Group 3) and stem‐like (Group 4) groups, where benign and preinvasive nodules tend to cluster. In contrast, *EGFR*‐driven (Group 1) and *EGFR*/*TP53* co‐mutation (Group 2) groups appeared relatively “cold” with fewer immune infiltrates (Figure 3A and B). The *EGFR‐*driven group (Group 1) exhibits a lack of T cell infiltration, especially CD8 T cell infiltration, which is consistent with the clinical observation that lung cancer patients with *EGFR* mutation tend to have an unfavorable response to PD‐1/PD‐L1 checkpoint inhibitors.^24^ In the *EGFR*/*TP53* co‐mutation group (Group 2), CD8 T cell infiltration was also low. Conversely, Group 2 showed higher levels of Th2 and Treg cell infiltration. A recent study revealed that the loss of *TP53* function in tumor cells results in the accumulation of Treg cells.^25^ Due to the inflammatory characteristics of benign nodules, immune cell infiltration in the inflamed group was significant, which aligns with the observation that this group demonstrated activation of numerous inflammatory pathways, such as IL‐2/STAT5 and IL‐6/JAK/STAT3. Interestingly, the stem‐like group (Group 4) exhibited very high natural killer (NK) cell infiltration (Figure 3B). We also identified a strong correlation between the WNT/β‐catenin pathway (Pearson's correlation coefficient (PCC) = 0.8) and a moderate correlation of the NOTCH pathway (PCC = 0.48) in relation to NK cells (Figure 4A and C). NK cells are a crucial component of the innate immune system, contributing to immunosurveillance.^26^ The Wnt/β‐catenin signaling pathway is involved in NK cell development and activates the development and function of natural killer T (NKT) cells.^27^ We postulated that NK cells infiltrating preinvasive nodules were part of the host's innate immunosurveillance mechanism to prevent invasion. To evaluate whether this association is specific to preinvasive nodules, we examined it using TCGA data. The results indicated that the correlation was moderate for both the WNT/β‐catenin (PCC = 0.37) and NOTCH (PCC = 0.56) signaling pathways (Figure 4B and D).

### 2.4 The DNA methylation characterization of lung nodules

**FIGURE 5:**
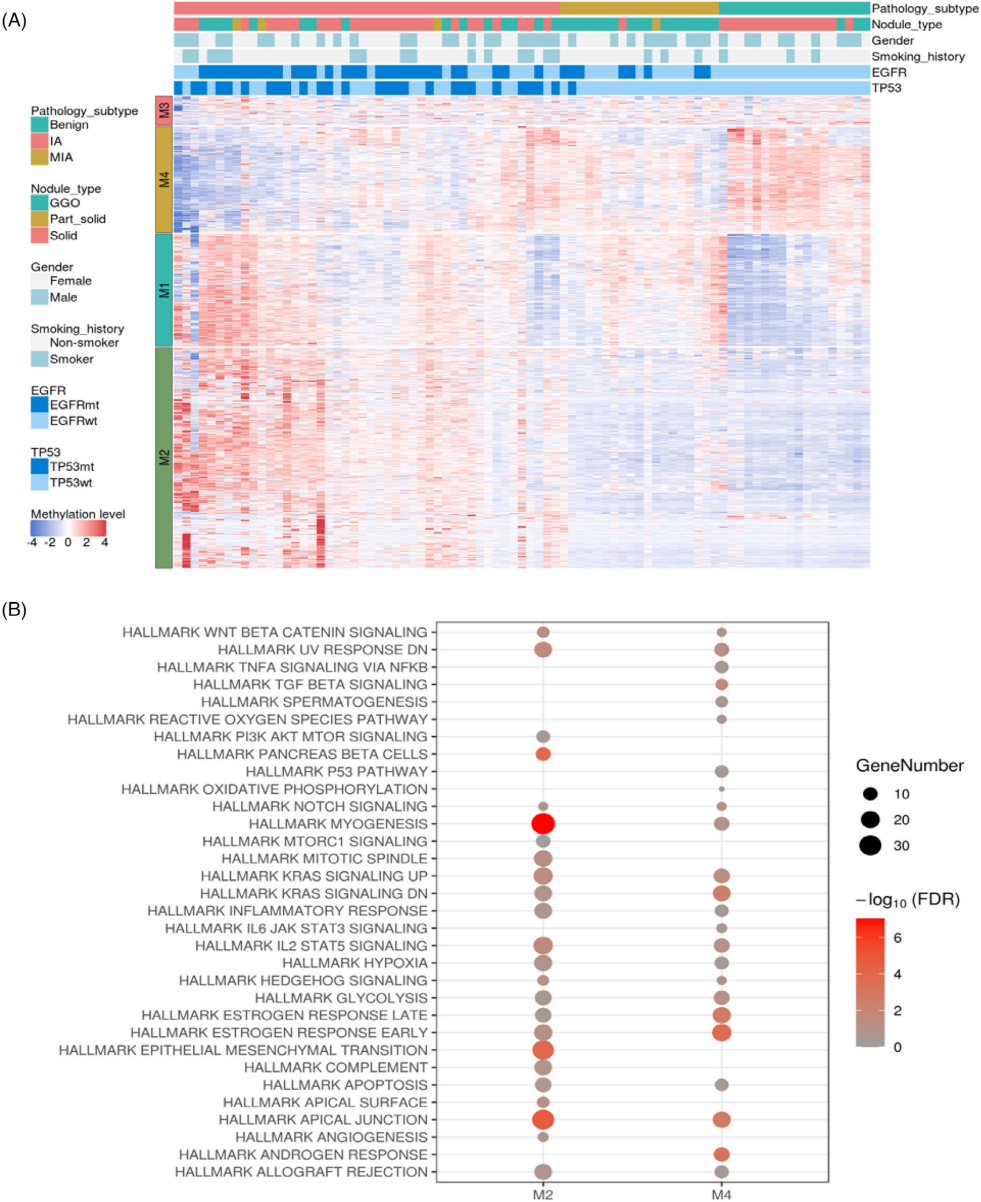
The DNA methylation characterization of lung nodules. (A) Phenotypes of DNA methylation cluster subgroups. The cluster assignments of patients are shown on the left. Red color indicates hypermethylation, and blue color indicates hypomethylation. (B) Pathway enrichment analysis for DNA methylation cluster subgroups M2 and M4.

To investigate the DNA methylation patterns in lung cancer, 83 DNA samples (comprising 18 benign, 19 MIA, 46 IA) were subjected to targeted DNA methylation sequencing. The data from a custom panel, containing 12,899 preselected probes, were clustered into four groups (M1, M2, M3, and M4) using unsupervised hierarchical clustering. Consequently, the M2 and M4 groups exhibited distinct methylation patterns across pathology subtypes. In the M2 group, markers were hypomethylated in benign and MIA nodules, while they were hypermethylated in IA nodules. In contrast, markers in the M4 group were hypermethylated in benign and MIA nodules but hypomethylated in IA nodules (Figure 5A).

To investigate gene functions, a hallmark gene set was utilized to conduct pathway enrichment analysis (Figure 5B). Genes within the M2 group were enriched in myogenesis, epithelial mesenchymal transition (*EMT*), IL2/STAT5 signaling, and the upregulation of KRAS signaling. EMT plays a role in tumor invasion and metastasis.^28^ IL2/STAT5 signaling regulates Treg cell differentiation and maturation.^29^ KRAS signaling was related to cell proliferation. Genes in the M4 group were enriched in ER and AR pathways, TGFβ signaling, and the downregulation of KRAS signaling. TGFβ signaling modulates tumor invasion and metastasis through EMT, angiogenesis, and immunosuppression.^30^ The enrichment of ER and AR pathways in M4 was consistent with the transcriptomics analysis of *EGFR*‐driven group (Group 1) in this study, further supporting the involvement of hormonal pathways in lung cancer development (Figure 3A).

### 2.5 The integration of gene expression and DNA methylation

**FIGURE 6:**
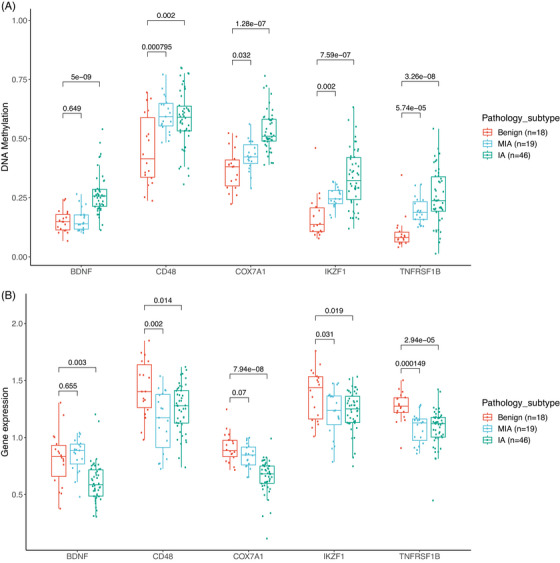
The integration of gene expression and DNA methylation in M2. (A) Comparison of DNA methylation levels between benign, MIA, and IA in the M2 group. (B) Comparison of gene expression levels between benign, MIA, and IA in the M2 group. ns, not significant, *p < 0.05; **p < 0.01; ***p < 0.001, Two‐sided Mann–Whitney *U* test.

There is evidence of both strong positive and strong negative correlations between gene methylation and gene expression.^31^ To pinpoint potentially relevant DNA methylation and gene expression alterations during the progression of lung nodules, an integrated analysis of DNA methylation and gene expression was conducted. Pearson's correlations were calculated to identify statistically significant inverse correlations between DNA methylation and gene expression of enriched genes from pathway analysis, yielding fourteen genes with DNA methylation alterations situated at the promoter regions. In the M2 group, five genes, including *BDNF* (brain‐derived neurotrophic factor), *CD48* (CD48 molecule), *COX7A1* (cytochrome c oxidase subunit 7A1), *IKZF1* (IKAROS family zinc finger 1), and *TNFRSF1B* (TNF receptor superfamily member 1B), displayed progressively increasing methylation levels, accompanied by corresponding decreases in gene expression levels from benign, MIA, to IA (Figure 6). The highest inverse correlation values in the M2 group were observed for *CD48* (−0.7), *COX7A1* (−0.65), and *TNFRSF1B* (−0.61). Our findings regarding *COX7A1* were consistent with recent studies showing that *COX7A1* had higher methylation and lower expression in the NSCLC cell line,^32^ which suppressed cell viability by downregulating tumor autophagy in vitro.^33^ Previous studies have demonstrated that *TNFRSF1B*, a gene associated with apoptosis, had mutations and lower expression in NSCLC patients,^34^, ^35^ which is supported by our findings. However, it has been reported that *BDNF* had a high expression level in lung squamous cell carcinoma and NSCLC, linked to tumor proliferation and invasion.^36^ *BDNF* can bind to TrkB receptors to activate ERK/CREB, PI3K/AKT, and NF‐kB pathways, ultimately enhancing tumor survival, proliferation, and migration.^37^ In contrast, our study revealed that *BDNF* had higher methylation and lower expression in IA, suggesting that *BDNF* may have distinct functions in early‐stage versus late‐stage lung cancers.

**FIGURE 7:**
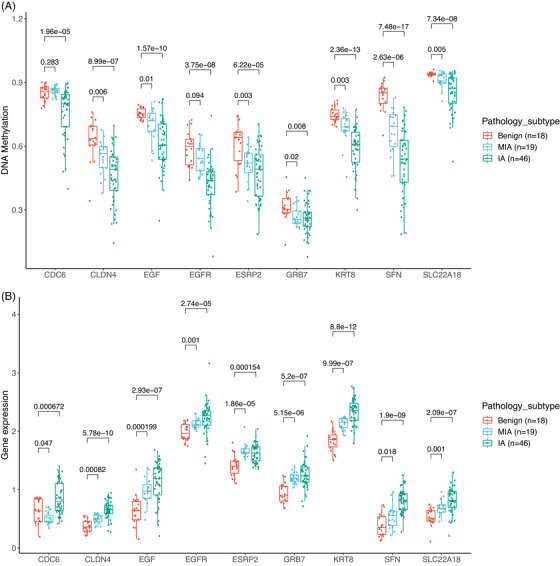
The integration of gene expression and DNA methylation in M4. (A) Comparison of DNA methylation levels between benign, MIA, and IA in the M4 group. (B) Comparison of gene expression levels between benign, MIA, and IA in the M4 group. ns, not significant, *p < 0.05; **p < 0.01; ***p < 0.001, Two‐sided Mann–Whitney *U* test.

Conversely, in the M4 group, nine genes, including *CDC6* (cell division cycle 6), *CLDN4* (claudin 4), *EGF* (epidermal growth factor), *EGFR*, *ESRP2* (epithelial splicing regulatory protein 2), *GRB7* (growth factor receptor bound protein 7), *KRT8* (keratin 8), *SFN* (stratifin), and *SLC22A18* (solute carrier family 22 member 18), exhibited progressively decreasing methylation levels and increasing levels of gene expression from benign, MIA, to IA (Figure 7). The highest inverse correlation values were observed for *EGF* (−0.78), *ESRP2* (−0.69), and *CDC6* (−0.63). *EGF* and *EGFR* had lower DNA methylation and higher expression in MIA and IA. Studies have suggested that the stimulation of *EGFR* by its ligand, *EGF*, may have pro‐tumorigenic effects, such as enhancing cell migration, with clinical implications.^38^ Upregulation of *EGFR* leads to activation of *EGFR* signaling and promotes tumor progression through *EGFR* downstream pathways, including PI3K/Akt/mTOR and RAS/MEK/ERK pathways.^39^, ^40^ Our findings emphasize the importance of the *EGFR* pathway and uncover a novel mechanism regulating this pathway during the neoplastic transformation from benign to IA. A previous study reported that NSCLC patients with strong overexpressing of *CDC6* had a significantly poorer prognosis compared to those with normal expression levels,^41^ consistent with our research. *KRT8*, a type II intermediate filament protein, is over‐expressed in various cancers, including lung cancer.^42^ While overexpression of *KRT8* has been attributed to DNA copy number gains,^43^ our results suggested that DNA hypomethylation may represent another mechanism for the upregulation of *KRT8*. Collectively, these genes hold promise as potential biomarkers for diagnostics and drug targets for the early interception of lung cancer.

## 3 DISCUSSION

Despite significant advancements in the genomic profiling of lung cancer in recent years, the genetic characteristics of benign, preinvasive, and invasive lung nodules remain largely unexplored. To the best of our knowledge, this study represents the most comprehensive multiomics investigation to date, aiming to elucidate the molecular pathogenesis of benign lung nodules and early‐stage lung cancers within the Chinese population.

In this study, we identified a high mutation frequency in genes such as *EGFR*, *TP53*, and *RBM10* in invasive nodules. Similarly, a previous investigation on pre/minimally invasive lung adenocarcinoma in the Chinese population revealed mutated genes like *EGFR*, *RBM10*, *BRAF*, *ERBB2*, *TP53*, *KRAS*, *MAP2K1*, and *MET*.^11^ Concordantly, the integrative proteomic characterization of LUAD revealed that clinical outcomes in early stage patients were linked to *EGFR* and *TP53* mutations.^44^ Our study corroborates these previous observations and demonstrates that exon 21 p.L858R and exon19 deletions were the predominant *EGFR* mutations. This study also supported previous reports on the presence of somatic mutations in the tyrosine kinase (TK) domain of *EGFR*, short deletions in exon 19, and point mutations (p.G719S, p.L858R, and p.L861Q) in exons 18 and 21.^45^, ^46^ Zhang's study reported the mutation rate of *EGFR* in AIS (31.3%, 5/16) was comparable to IAC (50%, 7/14) (*p* = 0.46),^47^ while Zhu's study found that the *EGFR* mutation frequency increased from AIS (33.3%) to MIA (50.8%) but remained stable from MIA to IAC (50.2%) stage.^48^ However, in our study, *EGFR* mutation frequency increased from AAH+AIS (16%), MIA (35%), to IA (73%), confirming the mutations of *EGFR* drive the transformation from preinvasive to invasive stages.

Interestingly, the TMB of nonsmokers was lower than that of smokers in both MIA and IA groups (Figure S5A), consistent with the previous report.^49^ Given that our cohort consisted of early‐stage lung cancer, we aimed to investigate the comparability of *EGFR* mutation frequency as a reference point by comparing the WES data of nonsmokers in our current cohort (early stage, MIA/IA) with the CHOICE cohort (late‐stage). The results showed that the mutation frequencies of the most commonly mutated genes, such as *EGFR* and *TP53*, were highly similar, with the exception of a few genes that exhibited notable differences (Figure S5B).

Abnormal regulations of alternative splicing can lead to the production of multiple transcriptional isoforms and diversified proteins, which often contribute to cancer development and progression. We identified frequent mutations in *RBM10* and *RBMX* among malignant nodules. *RBM10* mutations detected in LUADs disrupt the splicing of *NUMB* (NUMB endocytic adaptor protein), a NOTCH signaling pathway regulator, thereby promoting cell growth.^50^, ^51^ Truncation mutations of *RBMX* identified in lung cancer suggest it as a potential tumor suppressor gene.^52^, ^53^ *RBMX* mutations induced by cigarettes smoking may drive lung cancer development in smokers.^54^ Although no significant difference was observed between MUT and WT groups among pathological subtypes in terms of alternative splicing events for *RBM10* and *RBMX*, their significant roles in lung cancer progression warrant further investigation in larger cohorts.

Besides *EGFR* mutations, our study uncovered a novel mechanism for activating the *EGFR* pathway in lung cancer. Hypomethylation of *EGF* and *EGFR* leads to increased expression levels of these genes, presumably leading to higher activity of this vital pathway and driving lung cancer development. The *ER/AR* pathway was also upregulated in the *EGFR*‐driven group from the transcriptomic analysis. Estrogen and its receptors have been postulated to involve in the development and progression of lung cancer.^55^ A study reported that estrogen induces NSCLC cell proliferation by promoting *c‐Myc* and *Cyclin D1* expressions.^56^ Furthermore, estrogen signaling through *ERβ* regulates cell proliferation via the activation of *PI3K/IKK/NF‐κB*, *PI3K/AKT/Bcl‐XL*, and *RAS/RAF/MEK/ERK* signaling pathways.^57^ Estrogen has been reported to activate *ER* transactivation of the *EGFR* pathway and the nuclear localization of *Erβ*, which is significantly associated with *EGFR* mutations.^57^ The potential crosstalk between *EGFR* and *ER* signaling pathways has been documented in previous studies, leading to emerging clinical trials combining *EGFR* tyrosine kinase inhibitors (TKIs) with antiestrogen treatment.^56^, ^58^ Our study implies that the interaction of *EGFR* with *ER* signaling pathway may be pivotal in the tumorigenesis process and could aid in identifying lung cancer patients who would benefit most from the combination treatment.

Understanding the immune landscape within the tumor microenvironment during lung nodule progression is essential, as it may reveal the underlying molecular mechanism of susceptibility and resistance to immunotherapy and inform more effective strategies to enhance treatment outcomes. The RNA‐Seq data from the stem‐like group, enriched with preinvasive nodules, suggested a relationship between tumor stemness and the development of invasiveness. A recent study discovered that cancer stem‐like cells (CSCs) enhanced *PD‐L1* expression via *ALDH3A1* isozyme to promote immune evasion in lung tumors.^59^ Our study implies a potential link between NK cell infiltration and tumor stemness. NK cells can target and shape CSC‐nondifferentiated tumors, leading to the selection of differentiated tumor subpopulations with reduced proliferation rates, incapable of invasion or metastasis, and increased susceptibility to chemotherapy and radiotherapy.^60^, ^61^ We also found that Treg cell infiltration was associated with *TP53* mutations. The loss of *TP53* function in tumors results in the accumulation of Treg cells.^25^ Moreover, TP53‐null cancer cells promote *CXCL17* (C‐X‐C motif chemokine ligand 17) secretion, which is also associated with the expansion of immunosuppressive Treg cells.^62^ Based on these findings, remodeling the tumor immune microenvironment in patients at preinvasive stages prior to immunosuppression, may represent a beneficial therapeutic strategy.

Besides the aberrant DNA methylation of *EGFR*, our study revealed that other methylation changes also contribute to lung cancer initiation and development. Hypermethylation of genes such as *COX7A1*, *TNFRSF1B*, and *BDNF*, as well as hypomethylation of genes such as *CDC6* and *KRT8*, may be associated with lung cancer compared to benign nodules. *COX7A1* can suppress the viability of human NSCLC cells by regulating autophagy through the downregulation of *PGC‐1α* and upregulation of *NOX2*.^33^ As a critical mediator of *TNF/TNFR* signaling pathway, *TNFRSF1B* can promote apoptosis^63^ and necroptosis,^64^ which may explain the association between *TNFRSF1B* polymorphisms and the survival of lung cancer patients treated with chemoradiotherapy.^35^, ^64^ Apart from its role as a neurotrophins, *BDNF* can also enhance tumor survival, proliferation, and migration by activating the ERK/CREB, PI3K/Akt, and NF‐kB pathways via binding to the TrkB receptor.^65^ As a regulator of DNA replication, downregulation of *CDC6* can inhibit the DNA damage response activity of cancer stem‐like cells.^66^ *KRT8* has been identified as a target of cisplatin, and the KRT8/AKT signaling pathway plays a crucial role in lung cancer metastasis by regulating the function of cancer‐associated fibroblast in the tumor microenvironment.^67^ These genes may play a critical role in the initiation and development of cancer and could potentially be used as diagnostic biomarkers and drug targets for early‐stage lung cancers.

This study has several limitations that should be acknowledged. First, we included benign nodules instead of normal lung tissues, as benign nodules in CT images can be easily confused with malignant nodules, making it challenging to differentiate between them. Due to their frequent overdiagnosis and overtreatment in clinical practice, our goal was to distinguish the molecular characteristics of benign and malignant nodules. Second, since AAH and AIS are defined as precursor glandular lesions and are rarely surgically resected in clinical practice, it is regrettable that the AAH+AIS group was relatively small and lacked epigenetic data in the entire cohort. Third, we have not conducted in vitro*/*vivo experiments to investigate the molecular functions, such as the interaction between the *EGFR* pathway and the estrogen/androgen pathways. Future studies will further validate these findings in a more careful manner. Lastly, comparing different patient samples is influenced by various factors; however, investigating the molecular pathogenesis of nodule progression within an individual patient or within a single tumor is the best way to proceed. It is challenging to track a patient from the initial appearance of a lung nodule to the development of lung cancer and obtain tissue samples at various stages of the disease. Moreover, cases with multiple lesions containing benign, preinvasive, and invasive subtypes are uncommon. Therefore, larger cohorts with long‐term follow‐up are essential to validate our findings.

In summary, our study has presented the genomic landscape of lung nodules through an integrated analysis of whole exome sequencing, RNA‐Seq, and DNA methylation sequencing. This comprehensive approach enhances our understanding of the distinct molecular mechanisms underlying benign, preinvasive, and invasive lung nodules. Moreover, we have identified potential biomarkers for the early detection of lung cancer and drug targets for lung cancer treatments. Our study offers an unprecedented resource for unraveling cancer evolution and developing interception solutions.

## 4 METHODS AND MATERIALS

### 4.1 Participating patients and sample collection

This study enrolled patients with lung nodules (5–30 mm in diameter) detected by CT/LDCT scan at The First Affiliated Hospital of Guangzhou Medical University in China between 2018 and 2019. None of the patients had received any preoperative cancer treatments. FFPE tissue samples were acquired from subsequent surgical resections, and matched WBCs were also collected to subtract germline mutations. Both genders were included in the study, and smoking history information was gathered. The pathological results of all samples were determined based on surgically resected tissue sections according to the 2015 WHO Histological Classification of Lung Cancer.^68^ Written informed consents was obtained from all participants.

Sections of 5−10 µm in thickness were cut from the FFPE tissue samples of 432 patients with lung nodules. Approximately 8−10 mL of whole blood sample was drawn from each patient using a Cell‐Free DNA BCT blood collection tube (Streck, Inc. Cat# 218962). WBCs were separated immediately upon receipt of the whole blood samples according to the standard protocol and stored at −80°C until DNA isolation. Purity of tumor cells were estimated by two independent pathologists by reviewing Hematoxylin and Eosin (H&E) staining slides, with a minimum tumor cell content of 10% required for subsequent sequencing.

### 4.2 Nucleic acid isolations

gDNA and total RNA were simultaneously extracted from FFPE tissue samples using AllPrep FFPE DNA/RNA Kit (Qiagen, Cat# 80234) according to the manufacturer's instructions. gDNA from WBC samples was isolated using the DNeasy Blood & Tissue Kit (Qiagen, Cat# 69504). The concentrations of DNA and RNA were measured using the Qubit dsDNA HS Assay Kit (Thermo Fisher Scientific, Cat# Q32854) and Qubit RNA HS Assay Kit (Thermo Fisher Scientific, Cat# Q32855), respectively. The quality of DNA and RNA were assessed using the Agilent High Sensitivity DNA Kit (Cat# 5067‐4626) and the Agilent RNA 6000 Pico Kit (Cat# 5067‐1513) on a 2100 Bioanalyzer Instrument (Agilent Technologies, Inc.), respectively. Only samples with DV200 values (the percentage of fragments > 200 nucleotides) ≥ 30% were selected for RNA‐Seq.

### 4.3 Whole exome sequencing and RNA‐Seq

WES and RNA‐Seq were carried out by Mingma Technologies Co., Ltd. (Shanghai, China). In brief, 300 ng of gDNA from FFPE tissue or WBC samples were fragmented into 150−200 bp pieces (mean size) using a LE220‐plus Focused‐ultrasonicator (Covaris, Inc.). DNA libraries were prepared using SureSelectXT Reagent Kit (Agilent, Cat# G9611A) following the manufacturer's instructions. Whole exome enrichment was conducted using SureSelectXT Human All Exon V6 Kit (Agilent, Cat# 5190−8864). For RNA from FFPE tissue, 200 ng was converted into sequencing libraries using the TruSeq RNA Exome Kits (Illumina, Cat# 20020189, 20020490, 20020492/20020493, 20024145). The Resulting libraries were quantified using the Qubit dsDNA HS Assay Kit, and their size distributions were analyzed by Agilent DNA 1000 Kit (Cat# 5067‐1504) on a 4200 TapeStation System (Agilent Technologies, Inc.). Sequencing was performed in the 2×150‐bp paired‐end mode on the HiSeq X Ten or NovaSeq 6000 platform (Illumina, Inc.).

### 4.4 Targeted DNA methylation assay

Full details of DNA fragmentation, bisulfite conversion, and targeted methylation sequencing were described previously.^69^ Briefly, gDNA from 83 selected samples were fragmented into approximately 200 bp (peak size) using an M220 Focused‐ultrasonicator (Covaris, Inc.) following the manufacturer's instructions. 50 ng of purified, fragmented gDNA was used for the subsequent bisulfite conversion step. Bisulfite conversion was carried out using the EZ DNA Methylation‐Lightning Kit (Zymo Research, Cat# D5031) according to the manufacturer's protocol. The AnchorIRIS library preparation technology was employed for targeted methylation analysis. AnchorIRIS prehybridization library construction was performed using the AnchorDx EpiVisio Methylation Library Prep Kit (AnchorDx, Cat# A0UX00019) and AnchorDx EpiVisio Indexing PCR Kit (AnchorDx, Cat# A2DX00025). Target enrichment was performed using AnchorDx EpiVisio Target Enrichment Kit (AnchorDx, Cat# A0UX00031). A custom‐made lung cancer methylation panel (LC Panel), which includes 12,899 preselected probes enriched for lung cancer‐specific methylation regions, was utilized in the present study.^70^ After probe hybridization, the resulting libraries were sequenced in the 2 × 150‐bp paired‐end mode on the NovaSeq 6000 platform.

### 4.5 Data quality control

We used the default parameters of the FastQC (version 0.11.2) (http://www.bioinformatics.babraham.ac.uk/projects/fastqc/) software to perform quality control (QC) on all samples. Skewer (v0.2.2)^71^ software was applied to remove splice sequences from the sequencing platform raw data (Fastq). Sequences with a length of at least 75 bp were filtered by the splice removal process, and the resulting filtered data (clean reads) were used for downstream bioinformatic analyses. The exome capture efficiency and targeted region coverage were evaluated across the entire sample set. Lung nodules were sequenced at a mean targeted region coverage of 417×, with 97.7% coverage of ≥20×; WBCs were sequenced at a mean targeted region coverage of 215×, with 96.7% coverage of ≥20×, qualifying for subsequent variant analysis. The quality scores of 30 (%) for RNAseq was greater than 93.5. The average sequencing data amounts were 62.3 M, the average clean reads obtained after removing splices and low quality were 62.2 M, the average clean rate was 99.2%, and the average mapping rate was greater than 95%. For DNA methylation sequencing, the bisulfite conversion efficiency was greater than 99.2%, and the mean targeted region coverage was 785×, with 99% coverage of > 30×.

Three samples were excluded from further analysis due to low coverage in WES, two samples due to low coverage in RNA‐seq, and five samples due to a mismatch between tumor and normal samples. Therefore, 422 patients were included in the final analysis. Among them, 422 had qualified WES data, 228 had qualified RNA‐seq data, and 226 had both WES and RNA‐seq data (paired). For DNA methylation sequencing, 20 benign and 80 malignant samples (including 20 with *TP53* mutations, 20 with *EGFR* mutations, 20 with *TP53* & *EGFR* co‐mutations, and 20 without mutations) were included. Seventeen samples were excluded due to failing sequencing QC. Finally, 83 samples had qualified DNA methylation sequencing data.

### 4.6 Somatic variant identification

After removing adapters and low‐quality reads, the Sentieon (version 201911)^69^ pipeline with default parameters was implemented to process the following steps sequentially: reads alignment to the human genome reference assembly hg19 using the Burrows–Wheeler Aligner (BWA) algorithm and sorting, removal of PCR duplicates, InDel realign, base quality score recalibration, somatic mutation calling including single nucleotide variations (SNVs) and short insertion/deletions (indels), and variant quality score recalibration. During the mutation calling stage, the reads from the tumor sample were compared with the paired blood sample from the same patient to generate the somatic mutation list. Somatic mutations were then filtered and retained only with the variant allele frequency (VAF) ≥ 0.05 and supported by at least three reads. They were further annotated using the Variant Effect Predictor (VEP) package.^70^

### 4.7 Tumor mutation burden (TMB)

TMB was defined as the total number of somatic nonsynonymous mutations (SNVs or indels) in the tumor exome for each patient. The number was divided by the total size of the targeted regions to calculate the TMB score in counts/Mb. The Agilent SureSelect Human All Exon V6 kit was used, and its estimated total targeting size (exome) is 38 Mb. TMB was regressed on each gene to test for a statistical association between genes and TMB. Genes were identified according to the following criteria: correlation |*r*| > 0.4 and false discovery rate (FDR) < 0.05.

### 4.8 Copy number variation (CNV)

CNVkit v0.8.5 (https://cnvkit.readthedocs.io/en/stable/index.html) was used to analyze the copy number variation. In particular, for tumor/normal pairwise analysis, all normal samples were pooled together and then each tumor sample was compared with the normal pool to obtain tumor‐specific CNV, with filters of |log2ratio| ≥0.4 and bin number ≥ 5.

### 4.9 Splicing event counting

After assigning transcripts, alternative splicing events were identified using vast‐tools. Percent Splice In (PSIs) calculated by vast‐tools was then filtered using the cutoff psi > 80 (for IR, Alt5, Alt3) or psi < 20 (for EX) to count event number for each splicing type. Only high‐frequency splicing events (occurring in ≥ 10 samples) were preserved. The total splicing event number of a sample is the sum of the event number for each splicing type. The splicing gene list was curated based on the mRNA splicing gene set from Reactome.^72^

### 4.10 Differential gene expression analysis

RNA‐seq reads were mapped to human genome reference assembly hg19 using STAR (version 020201).^73^ The raw read counts were then normalized by log2‐counts per million normalization. The list of differentially expressed genes between groups was generated using DESeq2.^74^ Biological pathway enrichment was performed using the Kyoto Encyclopedia of Genes and Genomes (KEGG) and MSigDB hallmark gene sets. The FDR was calculated using the Benjamini and Hochberg (BH) false discovery rate (FDR) algorithm. We used an FDR ≤ 0.05 as the criterion to select significantly enriched KEGG pathways.

### 4.11 KEGG pathway enrichment analysis

The KEGG pathway database was used to perform pathway enrichment analysis. ClusterProfiler was used to obtain the latest online database and conduct an over‐representation test with the “enrichKEGG” function.^75^

### 4.12 Clustering analysis of ssGSEA scores based on hallmark gene set

The single sample gene set enrichment analysis (ssGSEA) scores of each sample based on Hallmark 50 gene sets were calculated, and then unsupervised hierarchic clustering of samples was performed based on the Hallmark ssGSEA scores to generate subgroups using the seaborns (version 0.11.2) package. The clustering result was then visualized by matplotlib in Python, and the immune cell ssGSEA scores corresponding to each sample were also plotted in the clustering heatmap. The Hallmark gene set used here was downloaded from the Gene set enrichment analysis (GSEA) molecular signatures database (gsea‐msigdb.org).

### 4.13 Immune cell signature score

RNA‐seq reads were assembled using StringTie2 (version 1.3.5), and FPKM and TPM data matrices were generated. TPM data were used to calculate the immunopheno score using the immunophenogram R package.^76^ FPKM data were used to calculate the immune infiltration score using the ssGSEA method.^23^ Infiltration cell types were clustered by heatmap analysis with the seaborns package and visualized by matplotlib in Python.

### 4.14 DNA methylation analysis

Sequencing adapters and three prime low‐quality bases were trimmed from raw sequencing reads using fastp (v0.19.6) software.^77^ In sillico converted hg19 was used as reference genome and cleaned reads were mapped to reference genome using Bismark (v0.18.2) software.^78^ Aligned reads were evaluated by Picard (v2.5.0) for metrics that measured the performance of target‐capture based bisulfite sequencing assays (http://broadinstitute.github.io/picard). The “bismark_methylation_extractor” tool was used to calculate methylation level for each targeted CpG site.

### 4.15 Differential methylation analysis

Differential methylation analysis was conducted using R package DSS (version 2.14.0).^79^ Differentially methylated CpGs (DMs) were first identified (criteria: FDR < 0.05, delta > 0.05), and adjacent DMs were further merged into differentially methylated regions (DMRs). DMRs were intersected with protein‐coding genes (hg19 Ensembl (v75), *n* = 20,232) by using annovar.^80^

### 4.16 Unsupervised hierarchical clustering of DNA methylation datasets

The preselected 12,899 probes were used for unsupervised hierarchical clustering. The methylation level of each targeted regions was calculated as the ratio of the methylated CpGs and the total sequenced CpGs (sum of methylated and unmethylated CpGs). Before clustering, the methylation levels of each targeted region were Z‐score normalized. The R function “hclust” was used to perform hierarchical clustering with “ward.D2” as the clustering algorithm. GSEA and MSigDB with BioCarta gene set, oncogenic signature gene sets and Hallmark gene sets were also used for biological pathway enrichment analysis.

### 4.17 Statistics analysis

Unless specified otherwise, Pearson's chi‐square test was used for *p*‐value calculations between two categorical variables. The two‐sided Mann–Whitney *U* test was used for comparisons between two continuous variables. The most frequently mutated genes and their distribution across different clinical features, the significantly different mutation genes between groups, and the mutually exclusive or co‐occurring mutated genes were calculated and visualized using the MAFtools package in R.^81^ GSEA was performed using the GSEA software.^82^ Pearson's correlation analysis was employed to calculate the coefficient between gene expression and methylation data. The *p*‐value < 0.05 was determined to be statistically significant for all analyses.

## AUTHOR CONTRIBUTIONS

Wenhua Liang, Jianbing Fan, Bo Wang, and Jianxing He conceived the study. Wenhua Liang, Jianbing Fan, Bo Wang, and Peng Liang designed the experiments. Bo Wang, Jun Wang, Hui Li, and Zhiwei Chen performed the experiments. Wenhua Liang, Lixuan Lin, Bo Cheng, Shan Xiong, Jianfu Li, Caichen Li, Ziwen Yu, and Chunyan Li contributed to data acquisition. Peng Liang, Minhua Peng, Jinsheng Tao, and Jinwang Wei contributed to data visualization. Wenhua Liang, Minhua Peng, Bo Wang, Jinwang Wei, and Jinsheng Tao wrote the manuscript. Jianxing He, Jianbing Fan, and Zhiwei Chen contributed substantially to the development of this manuscript. All authors reviewed and approved the manuscript.

## CONFLICT OF INTEREST STATEMENT

Authors Minhua Peng, Jinsheng Tao, Jun Wang, Hui Li, Zhiwei Chen, Jianbing Fan, and Bo Wang are current employees of AnchorDx Medical Co., Ltd, or AnchorDx, Inc. Jinwang Wei is a current employee of Genomicare Biotechnology (Shanghai) Co., Ltd and Shanghai CreateCured Biotechnology Co., Ltd. All other authors declare no competing financial interest.

## ETHICS STATEMENT

This study was approved by the Ethical Committees of The First Affiliated Hospital of Guangzhou Medical University (No. 2021−145). Written informed consents was obtained from all participants.

## Supporting information

Supporting Information

## Data Availability

AnchorDx Medical Co., Ltd provides access to the study protocol, the statistical analysis plan, the clinical study report, and all individual participant data except genetic data with academic researchers. Access is provided after a proposal has been approved by an Independent Review Committee identified for this purpose and after receipt of a signed Data Use Agreement. Proposals should be directed to contact-us@anchordx.com.
