# Supplementary material for: Development of a genome atlas for discriminating benign, preinvasive, and invasive lung nodules
Source: MedComm (2020). 2024 Jul 19;5(8):e644. doi: 10.1002/mco2.644 (PMC11258453; doi:10.1002/mco2.644)
Supplement: Supplementary file 1 — Supporting Information [file MCO2-5-e644-s001.docx]

**Online Data Supplement**

**Development of a Genome Atlas for Discriminating Benign, Pre-invasive, and Invasive Lung Nodules and Characterization of Their Molecular Pathogenesis in the Chinese Population**

Peng Liang, PhD; Minhua Peng, PhD; Jinsheng Tao; Bo Wang; Jinwang Wei, PhD; Lixuan Lin; Bo Cheng; Shan Xiong; Jianfu Li; Caichen Li; Ziwen Yu; Chunyan Li; Jun Wang; Hui Li; Zhiwei Chen, PhD; Jian-bing Fan, PhD; Jianxing He, MD; Wenhua Liang, MD

[Figure S1. 2](#_Toc129958730)

[Figure S2. 3](#_Toc129958731)

[Figure S3. 4](#_Toc129958732)

[Figure S4. 5](#_Toc129958733)

[Figure S5. 6](#_Toc129958734)

[Figure S6. 7](#_Toc129958735)


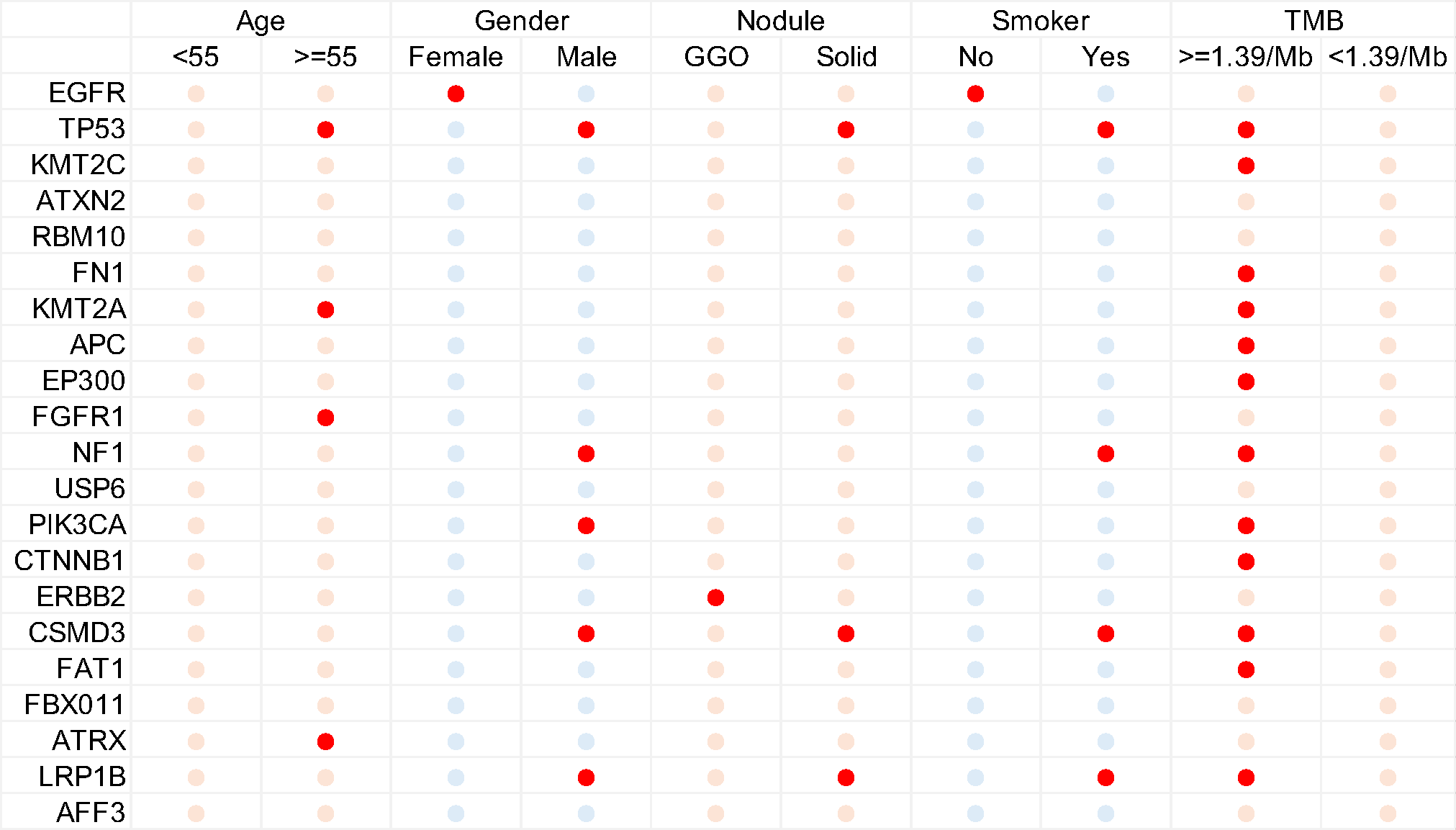


# **Figure S1.**

Correlation of somatic mutations and clinical characteristics.


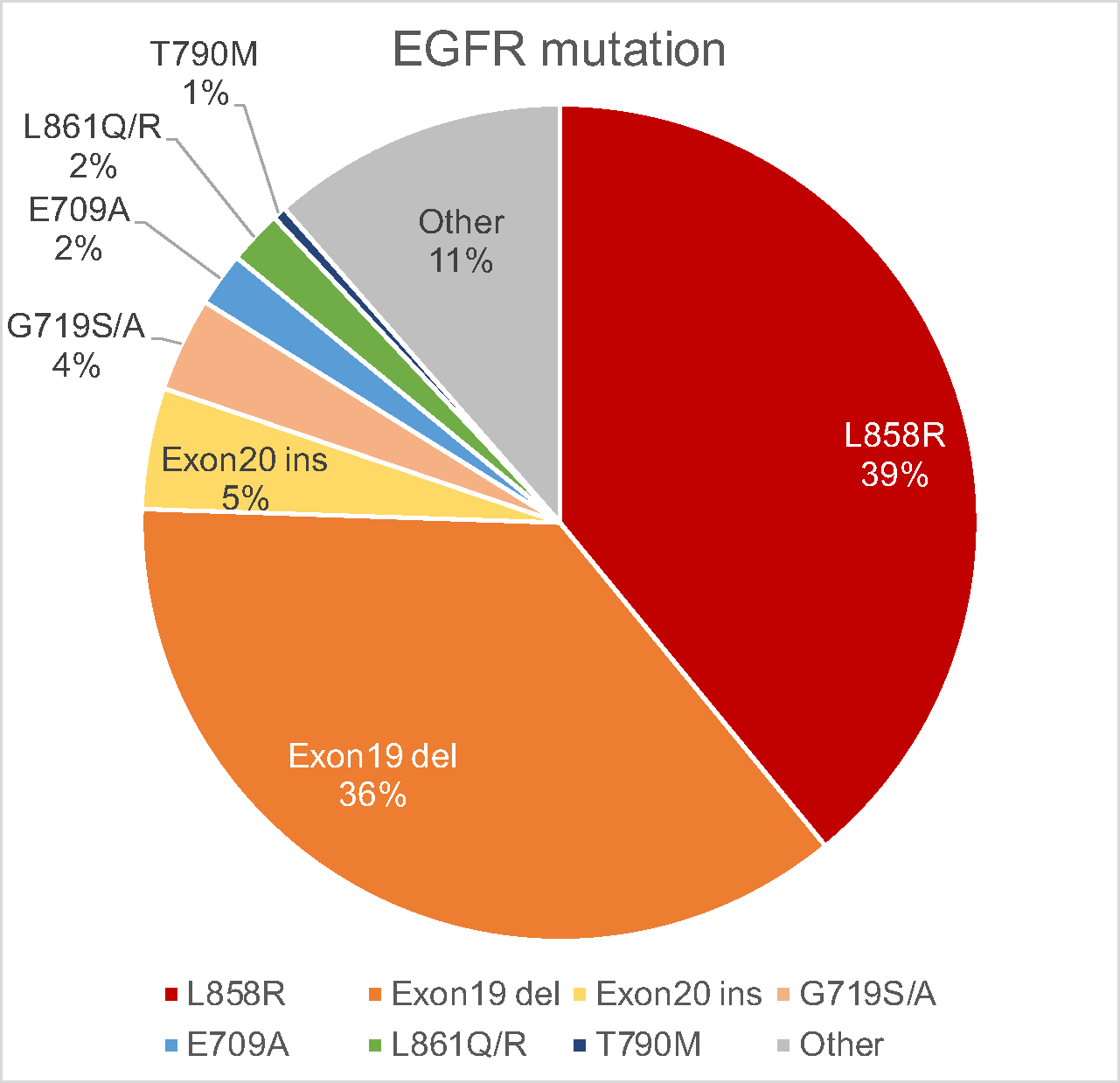
A


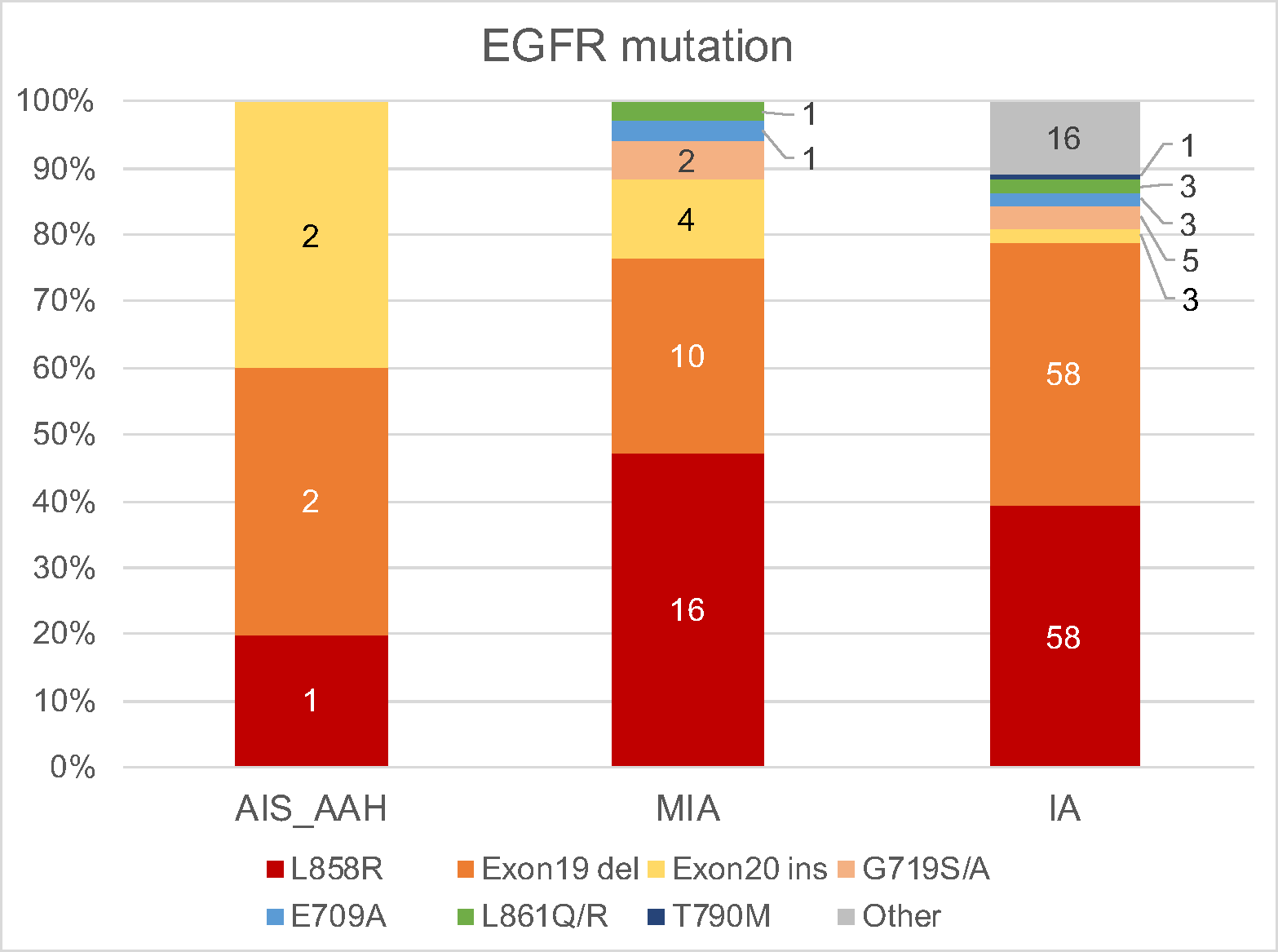
B

# Figure S2.

(A). The pie plot shows the proportion of different types of EGFR mutations.

(B). The bar plot depicts the number of different types of EGFR mutations in AIS_AAH, MIA, and IA, respectively.

#
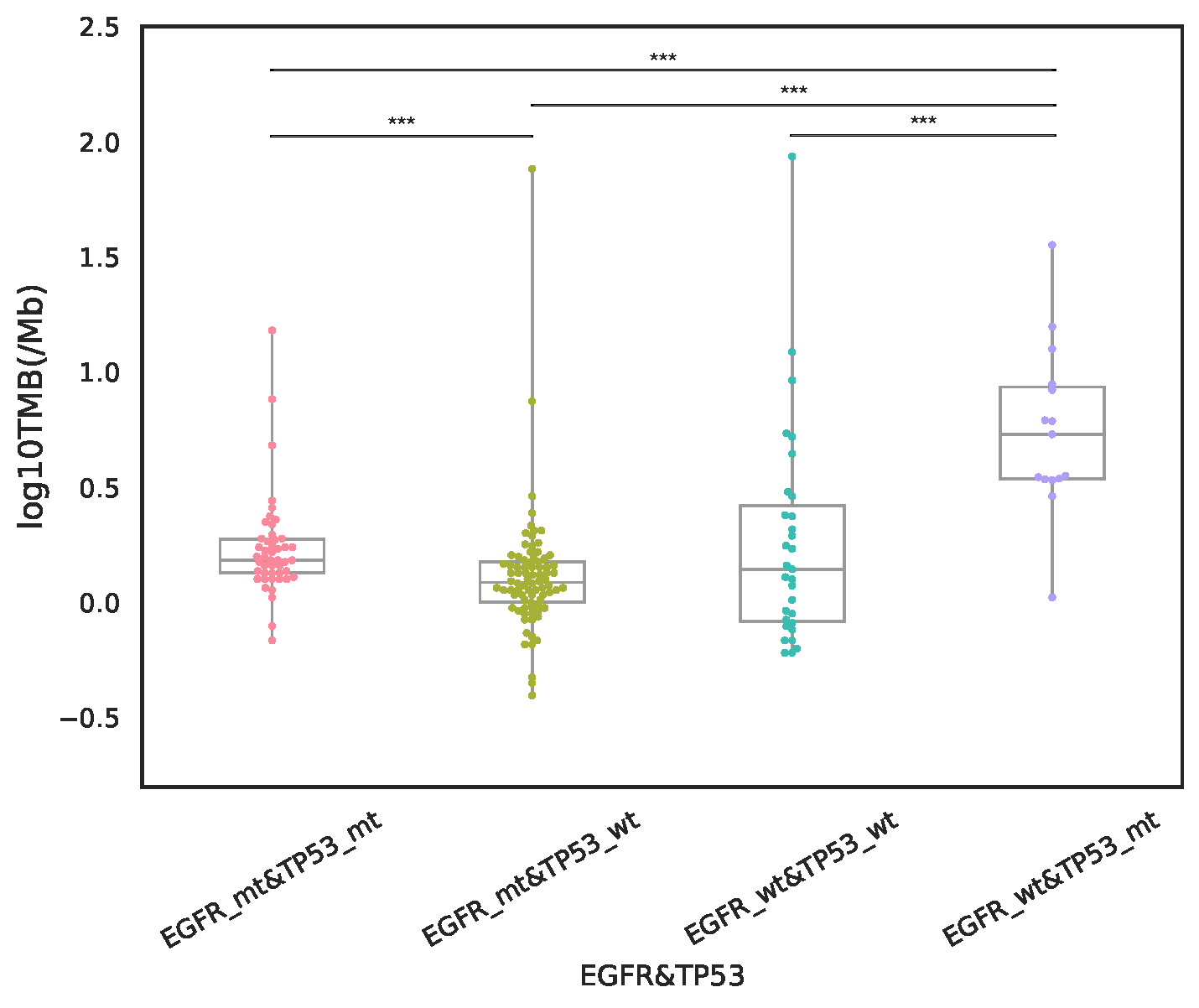
Figure S3.

Comparison of EGFR and TP53 WT/MUT status in IA nodule according to TMB.

ns: not significant, * p < 0.05; ** p < 0.01; *** p<0.001, Two-sided Mann Whitney U Test.


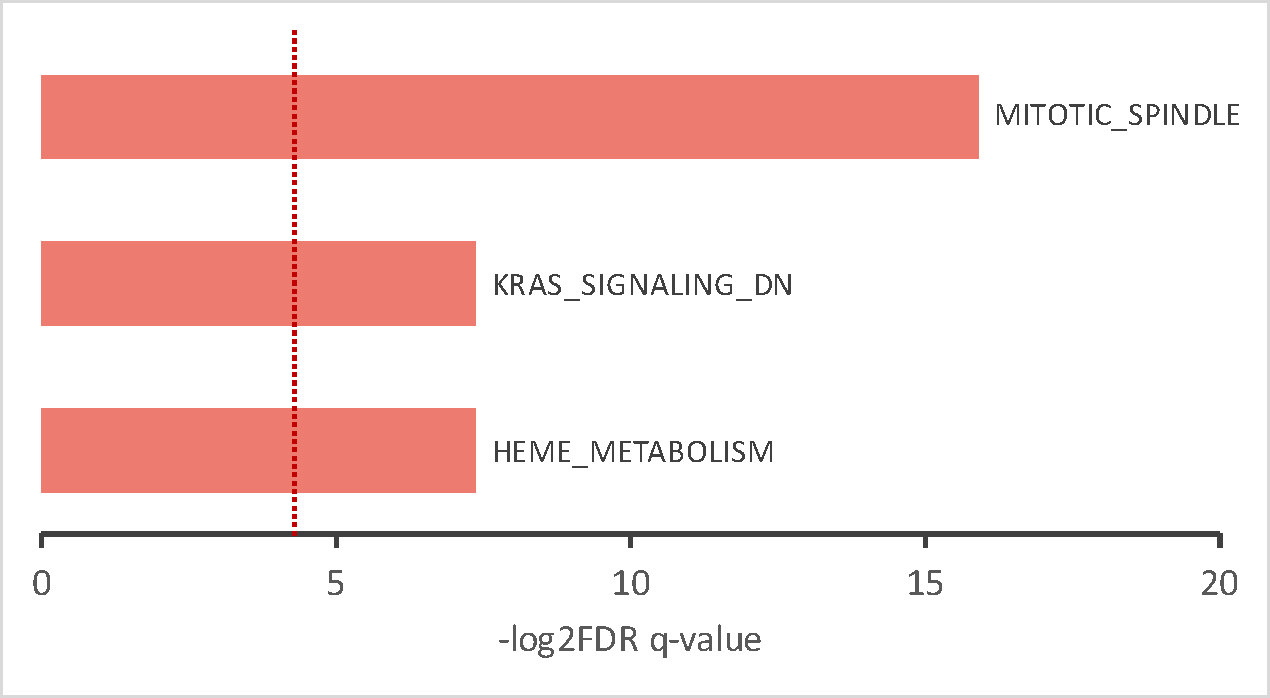
A


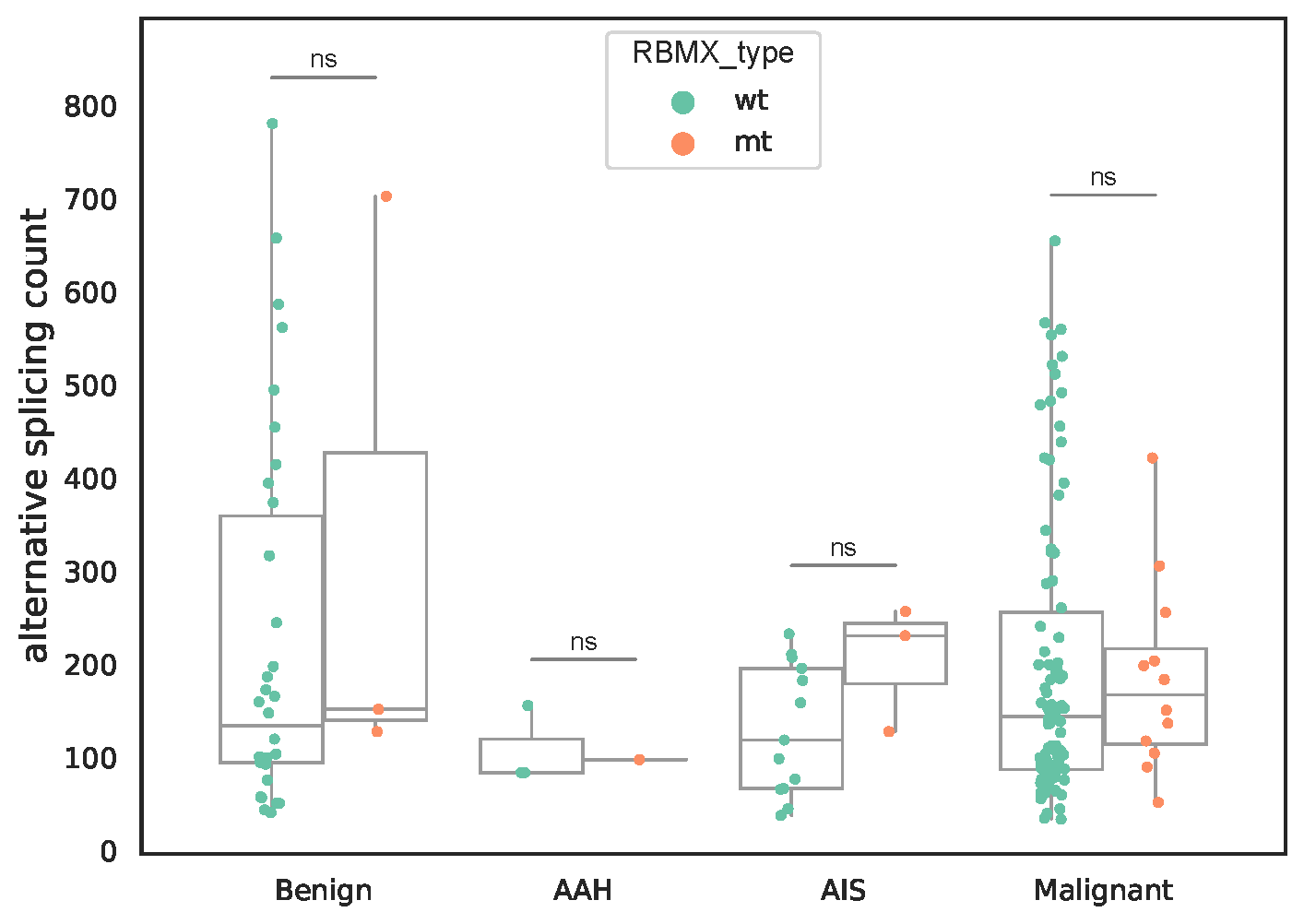

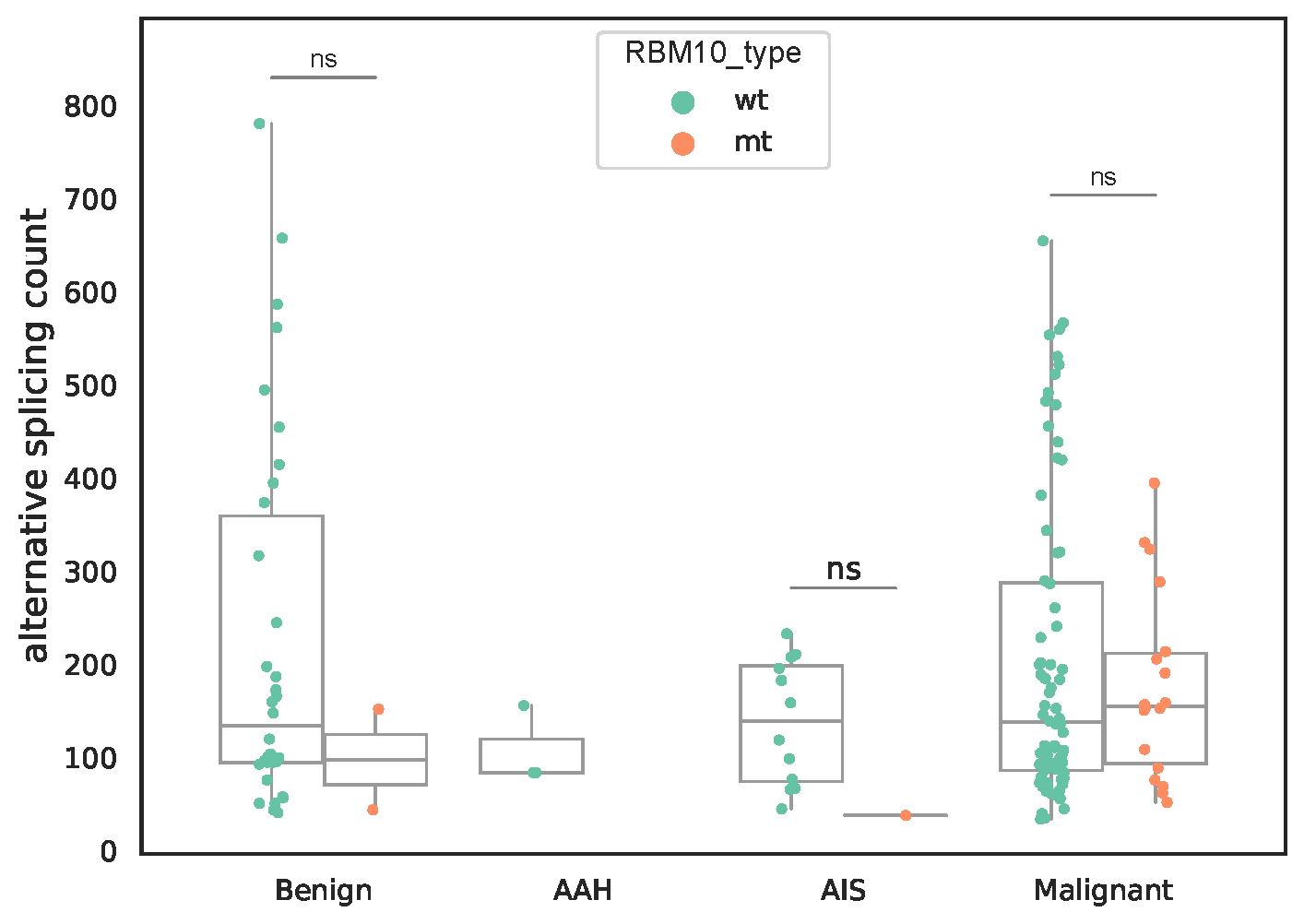
B C

# Figure S4.

(A). Pathway analysis of mutated genes with slicing events with false discovery rate (FDR) q value < 0.05.

(B). Alternative splicing events in each pathological subtype of nodules with WT (any splicing mutation excluded) or RBM10 mutation.

(C). Alternative splicing events in each pathological subtype of nodules with WT (any splicing mutation excluded) or RBMX mutation.

ns: not significant, * p < 0.05; ** p < 0.01; *** p<0.001, Two-sided Mann Whitney U Test.


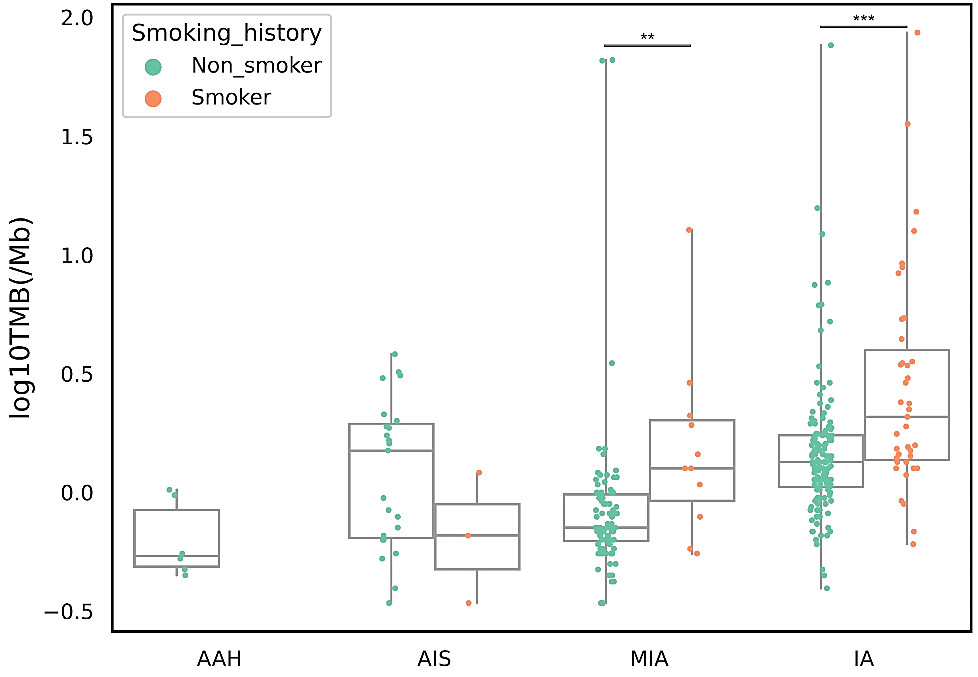
 A


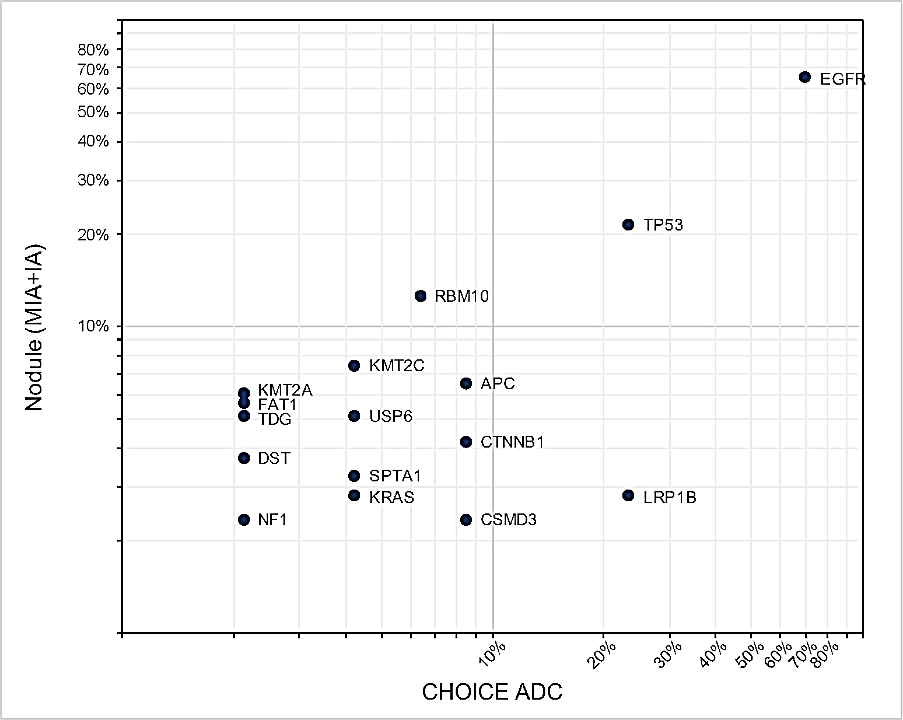
B

# Figure S5.

(A). Comparison of smoker and non-smoker in different pathological subtypes of nodules according to TMB.

(B). Mutation frequencies of the most common mutated genes in non-smokers in our cohort (early stage) and non-smokers in CHOICE cohort (late stage).

ns: not significant, * p < 0.05; ** p < 0.01; *** p<0.001, Two-sided Mann Whitney U Test.

# Figure S6.

Oncoplot depicting functional annotation of mutated genes. The genomic variations were annotated against a collection of comprehensive functional annotation databases using oncokb-annotator, to help understand and prioritize the SNVs and INDELs for further studies. Each row represents a gene and each column represents a single sample, with samples grouped by clinical classification. The barplot above represents the TMB of each sample. The barplot on the right represent the type of functions detected in each gene. Nodules samples were also labeled using 4 types of clinical data. (1) Pathological subtypes. (2) Nodule type. (3) Smoking history. (4) Gender.
